# Ag Nanorods-Based Surface-Enhanced Raman Scattering: Synthesis, Quantitative Analysis Strategies, and Applications

**DOI:** 10.3389/fchem.2019.00376

**Published:** 2019-06-04

**Authors:** Sumeng Zou, Lingwei Ma, Jianghao Li, Yuehua Liu, Dongliang Zhao, Zhengjun Zhang

**Affiliations:** ^1^State Key Laboratory of New Ceramics and Fine Processing, School of Materials Science and Engineering, Tsinghua University, Beijing, China; ^2^Institute for Advanced Materials and Technology, University of Science and Technology Beijing, Beijing, China; ^3^Department of Functional Material Research, Central Iron and Steel Research Institute, Beijing, China; ^4^Key Laboratory of Advanced Materials (MOE), School of Materials Science and Engineering, Tsinghua University, Beijing, China

**Keywords:** AgNRs-based substrates, SERS, synthesis, quantitative analysis strategy, detections, applications

## Abstract

Surface-Enhanced Raman Scattering (SERS) is a powerful technology that provides abundant chemical fingerprint information with advantages of high sensitivity and time-saving. Advancements in SERS substrates fabrication allow Ag nanorods (AgNRs) possess superior sensitivity, high uniformity, and excellent reproducibility. To further promote AgNRs as a promising SERS substrate candidate to a broader application scope, oxides are integrated with AgNRs by virtue of their unique properties which endow the AgNRs-oxide hybrid with high stability and recyclability. Aside from SERS substrates fabrication, significant developments in quantitative analysis strategies offer enormous approaches to minimize influences resulted from variations of measuring conditions and to provide the reasonable data analysis. In this review, we discuss various fabrication approaches for AgNRs and AgNRs-oxide hybrids to achieve efficient SERS platforms. Then, we introduce three types of strategies which are commonly employed in chemical quantitative analysis to reach a reliable result. Further, we highlight SERS applications including food safety, environment safety, biosensing, and vapor sensing, demonstrating the potential of SERS as a powerful and promising technique. Finally, we conclude with the current challenges and future prospects toward efficient SERS manipulations for broader real-world applications.

## Introduction

Surface-Enhanced Raman Scattering (SERS) is a promising analytical tool which provides identification and quantitative information of chemicals at trace levels based on their specific molecular vibrational fingerprints. Benefited from enormous advantages, such as non-destructive, high sensitivity, and time-saving, SERS was employed in several fields, including biosensing, food safety, environment safety, heterogeneous catalysis, and disease diagnosis, etc. (Zhao et al., 2015; Han et al., 2016; Chinnakkannu Vijayakumar et al., 2017; Tan et al., 2017; Kim et al., 2018). Therefore, SERS has attracted a lot of interests and many efforts have been devoted to develop ideal SERS substrates.

Generally, it is recognized that Raman signal enhancement is attributed to electromagnet enhancement (EM) and chemical enhancement (CM) (Kambhampati et al., 1998; Xu et al., 2000; Ding et al., 2017). EM is the dominant contributor originated from collective oscillation of localized surface plasmon resonance (LSPR) in metallic particles which are mainly coinage metals (Au, Ag, and Cu) (Willets and Van Duyne, 2007; Ding et al., 2016; Cardinal et al., 2017). Considering the correlation of LSPR to particles' shapes and sizes, thriving researches in substrate fabrications were focused on exploring SERS performance of particles with various shapes, such as nanospheres, nanorods, nanowires, nanoprisms, and nanocubes (Yang et al., 2015; Zhou et al., 2015; Chook et al., 2017; Ke et al., 2017; Wang et al., 2017). Among these, AgNRs proves to be a powerful SERS substrate candidate because it integrates the merit of the highest plasmonic resonance quality factor provided by Ag with the advantage of the tunable localized surface plasmon resonance (LSPR) peaks offered by the nature of nanorods (Rycenga et al., 2011; Li et al., 2017). Despite the rational preparation of AgNRs to realize its optimal SERS performance, intrinsic shortcomings of Ag nanoparticles including sensitivity degradation in severe measuring conditions and short shelf life still remain (Mai et al., 2012; Nuntawong et al., 2013; Bachenheimer et al., 2017). Moreover, the one-time use property of AgNRs makes substrates expensive which indicates the need to develop recyclable SERS substrates. These issues greatly restrict utilization of SERS as a universal technique. Fortunately, recent studies have shown that combining Ag nanoparticles with oxide was able to overcome these barriers by the virtue of unique properties of oxides (Kong et al., 2012; Wolosiuk et al., 2014; Chong et al., 2015).

On the other hand, considering that the appropriate analysis strategy is of crucial importance and the prerequisite to reach reliable chemical quantitative determinations, enormous quantitative analysis strategies were developed to minimize influences arising from variations of measuring conditions in order to obtain reliable data and further to provide the reasonable data analysis approach (Shen et al., 2015; Subaihi et al., 2016).

In this review, we cover the developments in AgNRs synthesis through oblique angle deposition method (OAD) to acquire sensitive, uniform, and reproducible SERS substrates. Then, approaches for AgNRs-oxide substrates preparation are introduced to achieve accurate control for the thickness of the oxide and to gain improvements in stability and recyclability with the merits of oxides. After surveying the recent progress in fabrication of AgNRs and AgNRs-oxide hybrids, in order to reach reliable SERS determinations, we then discuss three types of strategies which are commonly employed for chemical quantitative analysis: (1) external and internal method, (2) standard addition method, (3) multivariate data analysis. Later, to provide a clear understanding for readers, we further highlight SERS applications in the fields of food safety, environment safety, biosensing, and vapor sensing. At the end, we conclude with the challenges and potential opportunities in future SERS platform designs and utilizations.

## Synthesis and Properties of AgNRs-based Nanostructures

### Synthesis of AgNRs

**Figure 1:**
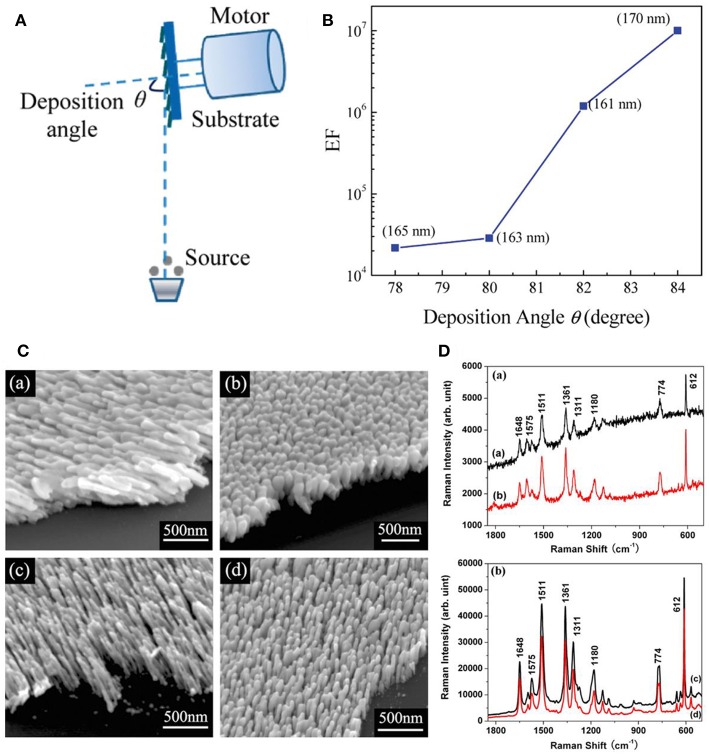
**(A)** Schematic illustration of nanostructure fabrication via OAD. **(B)** SERS enhancement factor of AgNRs as a function of deposition angle with nanorods length fixed at 165 nm. Reprinted with permission from Liu et al. (2010) Copyright (2010) ACS publications. **(C)** SEM images of AgNRs deposited (a) at 120°C without substrate rotation; (b) at 120°C and substrate rotation at 0.2 rpm; (c) at −40°C without substrate rotation; (d) at −40°C and substrate rotation at 0.2 rpm; **(D)** SERS spectra of R6G on (a) joined AgNRs shown in **C** (a,b); and **(b)** separated AgNRs shown in **C** (c,d). Reprinted with permission from Zhou et al. (2008). Copyright (2008) IOP Publishing.

High uniformity, superior sensitivity, and excellent reproducibility are always the most important targets for substrates preparations to allow SERS as a promising technique. Among numerous fabrication methods for AgNRs, oblique angle deposition (OAD) has been proved to be a straightforward approach to fabricate uniform and reproducible AgNRs which could also hold outstanding SERS sensitivity (Šubr et al., 2015). As shown in Figure 1A, OAD is a physical vapor deposition technique in which the incident vapor flux is deposited at a large incident angle θ (>70°) with respect to the substrate normal and Ag nanostructures with various morphologies and thicknesses can be readily obtained by simple control of the deposition angle and time (Liu et al., 2010; Ma et al., 2017). Specifically, substrates morphologies are dominated by a combination of atom shadowing effect and surface adatom diffusion effect (Negri and Dluhy, 2013). Taking account of the importance of AgNRs array's morphology on their SERS performance, critical parameters, such as incidence angle, temperature, and rotate rate during deposition process should be considered for optimizing SERS efficiency of substrates.

Slanted AgNRs is one of the commonest substrates fabricated by OAD technique, and their growth angle is expected to be positively correlated with the incident angle. Studies have shown that AgNRs tends to be well-separated and obtain stronger SERS sensitivity at larger deposition angle (Liu et al., 2010; Gao et al., 2018; Liao et al., 2018). Specifically, as shown in Figure 1B, Liu et al. further demonstrated that the enhancement factor (EFs) increases almost 1 order of magnitude as the deposition angle increases 2° by comparing EFs of AgNRs with similar length (Liu et al., 2010). Moreover, temperature exerts an impact on nanostructures' morphologies by influencing adatom diffusion. Hence, more considerations on temperature are required in nanostructure fabrication procedures, especially for materials with low melting temperature, such as Ag. Our group studied the effect of temperature on the morphology and SERS performance of AgNRs (Zhou et al., 2008). As shown in Figures 1C,D, well-separated AgNRs were obtained at a lower temperature, leading the better SERS performance. Singh et al. reported the cost-effective finding that AgNRs holding the maximum SERS sensitivity can be fabricated using less amount of Ag source at a lower temperature (Singh et al., 2012). In addition, the influence of rotate rate on the substrates morphology was investigated which showed that the formation of vertical nanorods were favorable at a higher rotation rate of substrate holder (Jen et al., 2015). Besides these experiment results, a simple hemisphere model was proposed to offer a theoretical route for analyzing the impact of crucial experiment factors in OAD method, such as incidence angle, incidence rate, substrate temperature, and substrate rotation rate on the morphology of nanostructures (Zhou et al., 2011b).

**Figure 2:**
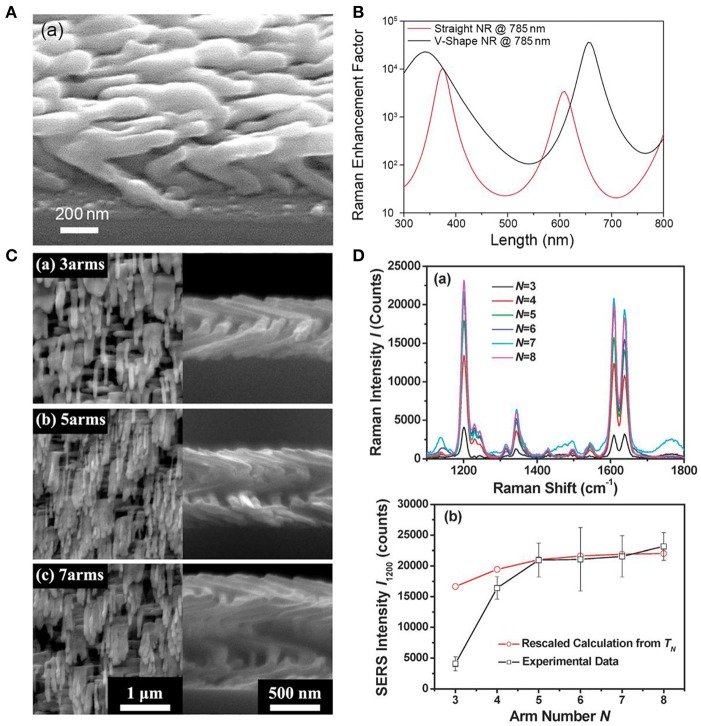
**(A)** SEM image of the V-shaped AgNRs fabricated by the GLAD method. **(B)** Numerical simulations of Raman EFs for the V-shaped AgNR and the straight AgNR as a function of AgNRs total length under a 785 nm laser. Reproduced with permission from Li et al. (2018). Copyright (2018) RSC publications. **(C)** SEM images of helical AgNRs with arm number at (a) *N* = 3, (b) *N* = 5, and (c) *N* = 7. **(D)** (a) SERS spectra of trans-1,2-bis (4-pyridyl) ethylene on helical AgNRs with various arm numbers. (b) Plots of experimental peak intensities (□) and calculations (°) as a function of arm number *N*. Reprinted with permission from Zhou et al. (2011a). Copyright (2011) RSC publications.

Although adjustments on the experiment conditions are able to provide straight AgNRs with the optimal SERS performance, the disadvantage that limited hotspots are generated in straight AgNRs still restricts the improvement in the substrate sensitivity. Therefore, more corners or curvatures are expected to be introduced within AgNRs array because they are regard as the hotspots which hold the extremely high electromagnetic field (Li et al., 2017). Recently, our group has been focused on the fabrication of multifold AgNRs via OAD in order to gain stronger SERS response. As shown in Figures 2A,C, AgNRs with multiple bends were successfully fabricated. Typically, after the fabrication of the first arm followed by a short cooling, the substrates holder was azimuthally rotated a certain angle to start deposition of the next arm. The numerical simulation was employed to compare EFs of V-shape and straight AgNRs with the same length, and the result (Figure 2B) showed that V-shape AgNRs exhibited stronger EFs in almost 90% cases under a 785 nm laser (Li et al., 2018). SERS performance of AgNRs as a function of the arm number *N* (*N* ≥ 3) was also investigated. As illustrated in Figure 2D, SERS response elevated rapidly with the increase of the arm number *N* when *N* ≤ 5 and the rate of enhancement slows down toward a plateau as *N* increases continuously (Zhou et al., 2011a). This trend should attributed to the combination result of “hotspots” amount, excitation light intensity reached to the “hot spot” layer, and optical absorbance of arm layers.

### Synthesis of AgNRs-Oxide Hybrid Composites

Despite advances in optimizing AgNRs fabrications and their SERS performances, fatal drawbacks that come from intrinsic properties of AgNRs, such as susceptible to severe measuring conditions including high temperature, etchant solutions, and oxidants, still remain a problem to be addressed (Park and Day, 1986; Alarifi et al., 2011; Nuntawong et al., 2013). Therefore, SERS substrates combine high sensitivity with great stability are desirable toward real life applications. On the other hand, the high cost of AgNRs substrates and one-time use only property hinder the widespread utilization of SERS technique. Given that incorporating AgNRs with oxides may bring unique properties to substrates by virtue of oxides (Li et al., 2017), in this section, we present the progress that have been made on the manipulation of AgNRs-oxides to achieve high stability and recyclability.

#### Oxide Coating

Although the oxide coating serves as a protective layer for AgNRs, the simultaneously increased distance between the plasmoinc active materials and analytes could lead to decreased EM enhancement for analytes. According to the studies, Raman signals of analytes within several nanometers from the plasmonic active materials are effectively boosted (Cardinal et al., 2017). Hence, precisely controlling the thickness of oxide coating is of significant importance to gain excellent SERS performance. Sol-gel method and atomic layer deposition (ALD) method are two approaches that are commonly employed to realize controllable thickness of oxide on AgNRs.

**Figure 3:**
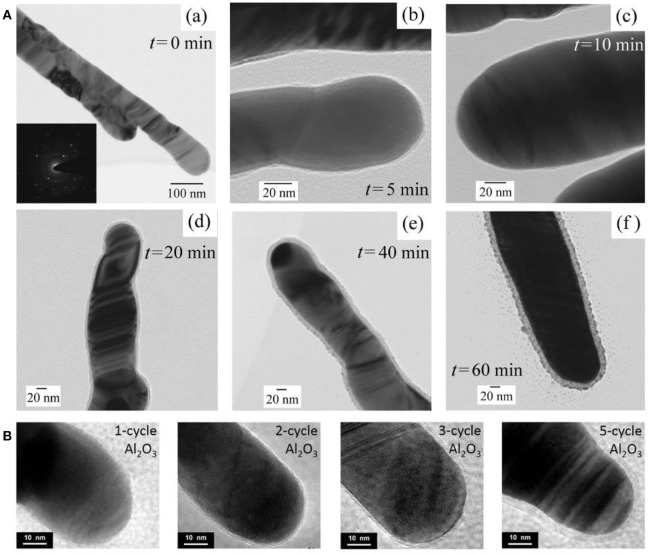
**(A)** HRTEM images of AgNRs coated by SiO_2_ as function of time by sol-gel method. (a) 0 min, (b) 5 min, (c) 10 min, (d) 20 min, (e) 40 min, (f) 60 min. Reprinted with permission from Song et al. (2012). Copyright (2012) ACS publications. **(B)** HRTEM images of AgNRs coated by Al_2_O_3_ with ALD cycles of 1, 2, 3, and 5. Reproduced with permission from Ma et al. (2015b).

Sol-gel method provides a facile way to synthesis ultrathin oxide layer, such as TiO_2_ and SiO_2_ (Song et al., 2012; Du et al., 2014, 2015). Taking SiO_2_ layer synthesis as an example, first, ethanol, DI water, NH_3_·H_2_O, and tetraethyl orthosilicate (TEOS) were mixed successively in a certain volume proportion to gain the SiO_2_ sol–gel precursor. Later, AgNRs modified with citric groups was added to this SiO_2_ sol–gel precursor solution and the thickness of SiO_2_ was readily controlled by changing the reaction time. Finally, the resultant AgNRs-oxide was removed from the solution once the reaction completes, followed by washing steps. Song et al. demonstrated that the uniform and porous SiO_2_ layer could be formed on individual AgNRs through this method (Song et al., 2012). As shown in Figure 3A, uniform SiO_2_ layers were successfully coated on AgNRs and a linear function between SiO_2_ layer thickness and the reaction time was further verified_._ However, in sol-gel method, SERS signals of citrate can be detected on prepared AgNRs-SiO_2_ substrates, resulting in the possible interference to the analytes detections.

On the other hand, ALD strategy serves as an effective way for oxide coating on AgNRs with desired thickness through self-limiting surface reactions (Wiedmann et al., 2012). Taking Al_2_O_3_ as an example, ALD process primarily contains these steps: (1) AgNRs were placed into an ALD deposition chamber with trimethyl-aluminum (TMA, maintained at 150°C) and water (maintained at 40°C) as precursors. (2) TMA was pulsed into the chamber for 20ms followed by 10s nitrogen purging. (3) Later, water was pulsed into the chamber for 10 ms followed by 20 s nitrogen purging. Then, the uniform and ultrathin oxide layer with various thicknesses could be obtained by repeating the cycle times of step (2) and (3). HRTEM images of AgNRs coated by Al_2_O_3_ layer with different ALD cycles were shown in Figure 3B, and uniform Al_2_O_3_ layers were formed on AgNRs with its thickness increased linearly vs. the cycle time (Ma et al., 2015b). Besides, a few pinholes could be observed on AgNRs-Al_2_O_3_ with 1 ALD cycle time which disappeared as cycle times increased.

The two approaches mentioned above both demonstrate the possibility of fabricating ultrathin oxide with acceptable sacrifice in sensitivity and the linear correlations between oxide layer thickness and the reaction time/cycle time were further established which imply the potential to fabricate reproducible and sensitive AgNRs-oxide as the SERS platform (John et al., 2010; Song et al., 2012). Following these advancements, fascinating properties of AgNRs-oxide in aspects of stability and recyclability were explored with the aim to expand SERS to a broader application scope.

#### Stability

Up to date, a library of diverse findings were reported in the field of the stability improvement for AgNRs through oxide coating, and they are usually in three aspects: (1) temporal stability, (2) chemical stability, and (3) thermal stability.

##### Temporal stability

**Figure 4:**
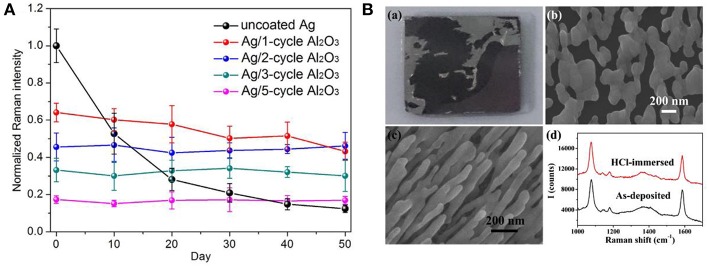
**(A)** Normalized Raman intensities of methylene blue at 1,622 cm^−1^ on AgNRs and AgNRs coated by various layers Al_2_O_3_ vs. storage time. Reproduced with permission from Ma et al. (2015b). **(B)** Morphologies and SERS performance of AgNRs and AgNRs-HfO_2_ after immersing into 10 mM HCl. (a) Images of AgNRs array on wafer. (b) SEM images of AgNRs. (c) SEM images of AgNRs-HfO_2_ array. (d) SERS spectra of 1 × 10^−5^ M 4-Mercaptobenzoic acid obtained from AgNRs-HfO_2_ before and after 10 mM HCl treatment. Reproduced with permission from Hou et al. (2016).

As shelf-time stability is the fundamental concern toward practical utilizations of SERS substrates, therefore, the mechanism for signal degradation of AgNRs in the ambient was studied by many researchers, leading a consensus that contaminants including sulfur, hydrocarbons, and oxygen were responsible for the SERS signals damping (Nuntawong et al., 2013; Bachenheimer et al., 2017). To pursue long temporal stability of substrates, Ag nanoparticles coated with oxides were estimated as SERS substrates. In the presence of the oxide layer, the contaminants diffusions from the air to the surface of AgNRs are effectively suppressed, resulting in great temporal stability improvement for substrates (Wolosiuk et al., 2014; Ma et al., 2015b; Zhao et al., 2016; Nguyen et al., 2017). For example, Nguyen et al. demonstrated that the thin shell of silica (~1.5 nm) enabled SERS activity of Ag@SiO_2_ nanocubes maintained at ~90% over 12 weeks (Nguyen et al., 2017). Moreover, Ma et al. investigated the SERS sensitivity of AgNRs-Al_2_O_3_ with different thicknesses as a function of the storage time (Ma et al., 2015b). As shown in Figure 4A, the Al_2_O_3_ layers with different thicknesses were fabricated by simply adjusting their cycle times of ALD. SERS response of AgNRs wrapped by 1 cycle Al_2_O_3_ layer decreased ~18% after 50 days in air, and SERS sensitivity of AgNRs coated with two or more Al_2_O_3_ cycles kept at the same level.

##### Chemical stability

The poor chemical stability of AgNRs in solutions mainly comes from their vulnerability to acids, halides and other etching chemicals that may impose the structural deterioration. To overcome this issue, chemical stabilities of Ag-oxides have been estimated by many researchers. For example, Du et al. reported that the oxide could serve as the anti-corrosion protective layer for Ag nanoplates and effectively retain their plasmonic property in salts, buffer, and oxidant solutions (NaCl, PBS, and H_2_O_2_) (Du et al., 2014). Ma et al. proved that 1 ALD cycle of Al_2_O_3_ layer was efficient to keep the morphology and SERS performance of AgNRs in NaCl solution (30 mM, 3 h) and H_2_O_2_ solution (2.2%, 0.5 h) (Ma et al., 2016a). Moreover, Hou et al. investigated the acid tolerance of AgNRs-HfO_2_ by immersing them into 10 mM HCl solution. As shown in Figure 4B, AgNRs suffered from violent corrosion by HCl. In contrast, the morphology and SERS performance of AgNRs-HfO_2_ remained the same, demonstrating the potential of AgNRs-HfO_2_ substrate to be utilized in acid solution (Hou et al., 2016).

##### Thermal stability

**Figure 5:**
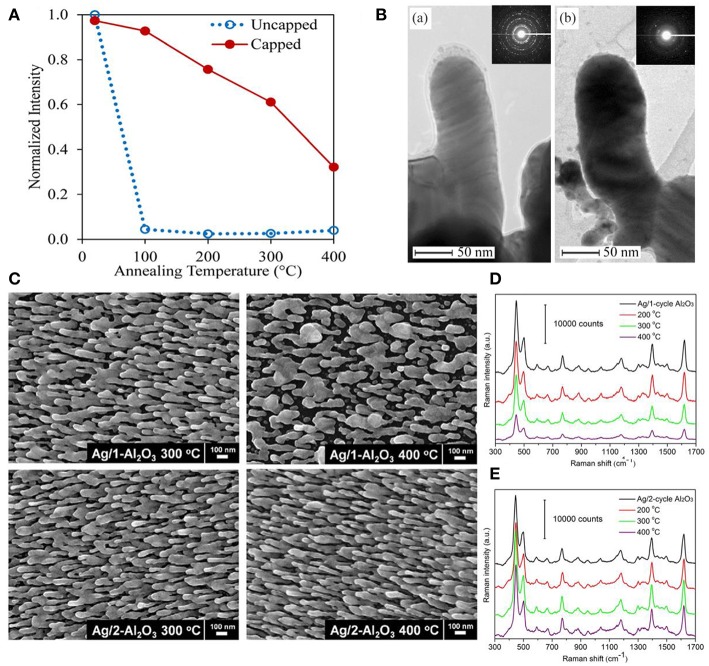
**(A)** Normalized SERS intensity on capped and uncapped AgNRs as a function of annealing temperature. **(B)** TEM images of TiO_2_ capped AgNRs after annealing at (a) 100°C for 10 min and (b) 200°C for 10 min. Reproduced with permission from Lou et al. (2014). Copyright (2014) AIP Publishing. **(C)** SEM images of AgNRs coated with Al_2_O_3_ layers at elevated temperatures. SERS spectra of methylene blue on AgNRs coated with 1 cycle Al_2_O_3_ layer **(D)** and 2 cycle Al_2_O_3_ layers **(E)** at elevated temperatures. Reproduced with permission from Ma et al. (2015b).

As SERS performances of substrates are highly correlated with their shapes and sizes, it is vital to preserve their geometric shape during detection conditions especially for low melting temperature materials, such as silver. Studies demonstrated that dramatic morphologies changes and devastating sensitivity degradation were observed on Ag nanoparticle substrates under high temperature and enhanced thermal stability could be effectively obtained by integrating oxides into SERS substrates (Mai et al., 2012; Pinkhasova et al., 2013; Lou et al., 2014; Ma et al., 2015b). For example, Lou et al. proved that capping TiO_2_ layer on AgNRs via PVD effectively protected AgNRs morphology up to at least 100°C (Figure 5A) (Lou et al., 2014). As shown in Figure 5B, they found that AgNRs started to coarsen once the TiO_2_ layer was no longer present under high temperature. Besides, the uniform oxide layer coated on AgNRs not only proves the effectiveness to improve substrates thermal stability, but also shows the potential to further enhance the thermal stability of substrates by increasing the oxide layer thickness. For example, as shown in Figures 5C,D, both the morphology and SERS performance of AgNRs coated by one cycle of Al_2_O_3_ layer started to change when the temperature reached to 300°C, while that of AgNRs coated by two cycles of Al_2_O_3_ layer remained the same even at 400°C (Ma et al., 2015b).

#### Recyclability

**Figure 6:**
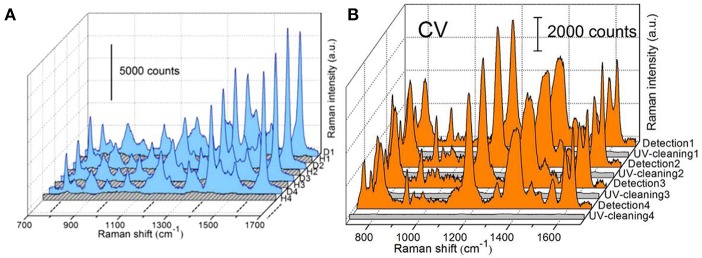
**(A)** SERS spectra of methylene blue (MB) on AgNRs-HfO_2_ during the repeated “detection (D)—heating (H)” process. Reproduced with permission from Ma et al. (2016b). Copyright (2016) ACS publications. **(B)** SERS spectra of crystal violet (CV) adsorbed on AgNRs-TiO_2_ during the repeated “detection-UV cleaning” process. Reproduced with permission from Ma et al. (2015a).

Although remarkable advancements in integrating oxide with AgNRs for the purpose of stability improvement, the cost of substrates emerges as another non-negligible issue which could limit the commercial utilization of SERS technique. To overcome this problem, the physical approaches including solvent washing and heat treatment as well as the chemical approach, such as photocatalysis were usually employed toward realizing substrates recyclability (Lin et al., 2012; Hou et al., 2015b; Ma et al., 2015a; Weng et al., 2016; Botta et al., 2018b). For AgNRs substrates, due to its susceptibility to heat and UV illumination, solvent washing becomes the common approach to remove adsorbed molecules from substrates. For example, Botta et al. examined the feasibility of reusing AgNRs through a simple de-ionized water washing (Botta et al., 2018b). They found that analytes molecules could be completely rinsed off from substrates and almost similar Raman signals re-appeared after analytes re-adsorbing on the substrates in the first three adsorption-desorption cycles. However, the reusability of AgNRs was found lost at the fourth repeated runs. Beside the solvent washing approach to remove analytes from substrates which should be universal to SERS substrates, the recyclability of AgNRs-oxide through heat treatment and photodegradation was investigated by our group. Ma et al. estimated the reusability of AgNRs-HfO_2_ through heat treatment (Ma et al., 2016b). As shown in Figure 6A, no Raman signatures of MB was observed after AgNRs-HfO_2_ were treated at 250°C for 30s which indicates their successful detachment from AgNR-HfO_2_ substrates. Later, MB signatures re-appeared on substrates through re-adsorbing without losing its intensity for as many as 30 repeated cycles, indicating super reusability of AgNRs-HfO_2_ through heat treatment by virtue of its robustness and thermal stability. Moreover, reusability of substrates obtained through photodegradation of analytes was estimated. AgNRs-TiO_2_ was employed due to the self-cleaning property of TiO_2_(Ma et al., 2015a). As shown in Figure 6B, AgNRs wrapped by 3 cycles of TiO_2_ layer via ALD proved to be recyclable within 4 repeated runs by UV-illumination, and no damage on SERS sensitivity was observed. Moreover, Fe_2_O_3_-Ag hybrid was also demonstrated as the reusable SERS substrates to be recycled 5 times without losing its sensitivity with the aid of visible light (Weng et al., 2016).

## Quantitative Analysis Strategy

Benefitting from the advancements in the field of substrates fabrication, SERS substrates that possess numerous superior properties, such as excellent sensitivity, reproducibility, stability, and cost saving, have been successfully engineered. This has driven the thriving research in the field of quantitative analysis strategies to achieve reasonable and accurate SERS measurement. These strategies include: (1) external and internal method; (2) standard addition method; (3) multivariate data analysis.

### External and Internal Method

External method is a straightforward approach for quantitative analysis which is based on the relationship between analytes concentration and its characteristic peak intensity/peak area. At submonolayer concentration, a linear correlation was always observed and thereby established as the standard curve which was utilized to predict analytes concentration (Zhu et al., 2016; Rekha et al., 2018). However, SERS intensities can be interfered with many factors in practical SERS measurements, such as laser power variations and environmental changes, leading an unreliable result (Bell and Sirimuthu, 2008). Therefore, the internal method was proposed to minimize the interference effect. In this method, an internal standard (IS) was introduced into the measurement system, generating its SERS signals with which SERS signals of analytes were able to calibrate. For example, Ren's group employed the characteristic band of phosphate backbone as the internal standard signal to calibrate signals from each base of DNA in order to achieve a reliable determination for DNA structure (Xu et al., 2015). Moreover, embedding internal standard molecules into core-shell nanoparticles emerges as another strategy to effectively correct the signal fluctuations of targets and this strategy proved to be efficient to provide reliable results by many researchers (Joshi et al., 2014; Shen et al., 2015).

### Standard Addition Method

**Figure 7:**
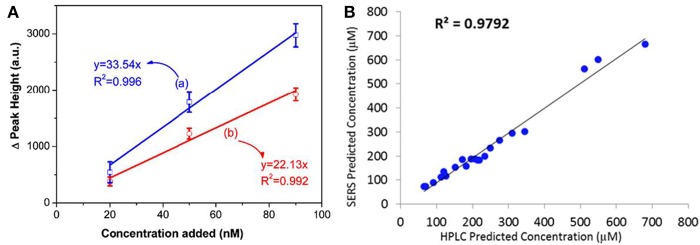
**(A)** Linear correlation between Raman peak intensity increment at (a) 365 cm^−1^, (b) 1379 cm^−1^ with the addition amount of 2-NaT ranging from 20 to 90 nM. Reproduced with permission from Zou et al. (2018). **(B)** The plot of comparison between results obtained from HPLC and SERS with the aid of SAM. Reproduced with permission from Westley et al. (2017). Copyright (2017) ACS publications.

The external and internal method depend on the well-established standard curve and a similar chemical component in the detection system as the standard solution is required to achieve accurate prediction of analytes. However, in practical applications, it is often hard to gain a prior knowledge concerning the testing solution which enables standard curve acquisition a difficult task. To overcome this problem, the standard additional method (SAM) was combined to the SERS analysis (Hidi et al., 2016; Westley et al., 2017). To utilize SAM, a series of analytes solutions with different concentrations were added into the sample whose composition is unknown. At trace levels, a linear correlation between the spiked analyte amount (*x*) and its characteristic peak/area intensity (*y*) should be observed which can be expressed as *y* = *mx* + *b*. In this equation, *b* represents the intensity of analytes initially existed in the sample and *m* is an indicator of intensity increment rate due to the addition of analytes. Conceptionally, the numerical value of *m* can be deduced from the linear relationship between the intensity increment of analytes with respect to its original value and the addition amount, and the numerical value of *b* is easily obtained by SERS measurement before the addition of analytes into the sample. Therefore, the initial amount of analytes in the sample can be obtained by the numerical value of *b* divided by *m*. For example, through establishing the correlation between the intensity increment and the addition amount, Zou et al. accurately predicted concentration of 2-naphthalenethiol (2-NaT) in the prepared binary solution (Figure 7A), and further employed this method for pollutants determinations in water (Zou et al., 2018). Westley et al. utilized this method to quantify uric acid in urine samples and compared results with high performance liquid chromatography (HPLC). As shown in Figure 7B, a good agreement was observed, indicating the reliability of this method (Westley et al., 2017).

### Multivariate Date Analysis

Quantitative analysis strategies mentioned above use some characteristic peaks of analytes, which may fail to predict analytes concentrations in the case when peaks are overlapped and thereby make the accurate intensity acquisition a difficult task. This situation is common especially in multi-component systems. Therefore, more sophisticated algorithms were developed to allow multivariate date analysis by exploiting the whole spectrum or a subset of the spectral features.

**Figure 8:**
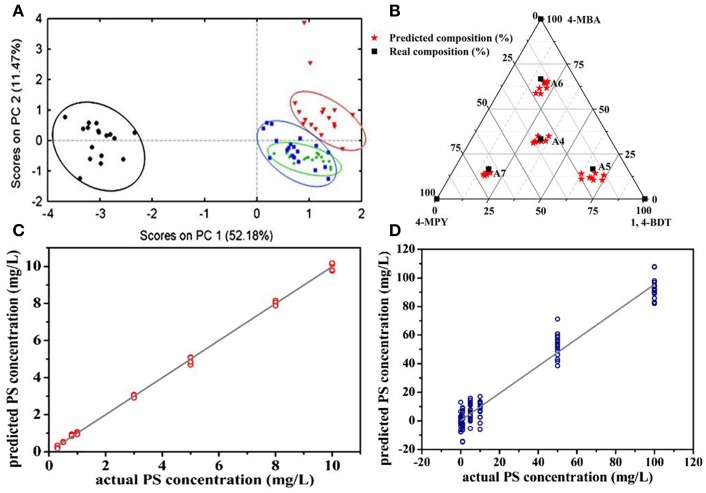
**(A)** The plot of PCA scores obtained from SERS spectra of four different respiratory syncytial virus strains. Reproduced with permission from Shanmukh et al. (2008). Copyright (2008) Springer Nature. **(B)** The plot of real compositions of component in ternary solution vs. that obtained by correcting PCA predictions. Reproduced with permission from Zou et al. (2017). **(C)** The plot of PS concentration predicted by the PLSR model vs. their actual concentration. **(D)** The plot of PS concentration in binary solution predicted by the PLSR model vs. their actual concentration. Reproduced with permission from Hou et al. (2016).

Principle component analysis (PCA) is a well-established chemometrics strategy for multivariate data analysis with principles to reduce the dimensionality of multivariate data and to preserve most of the valuable variance (Goodacre et al., 2018). The spectra data are converted into a set of loadings and scores and the first score (PC1) contains the most natural variance of the data followed by the one which is of less importance. Based on the PCA scores, PCA is widely employed to differentiate different molecules in the field of biosensing (Shanmukh et al., 2008; Chen et al., 2012, 2016; Botta et al., 2018a). For example, Shanmukh et al. demonstrated that three different respiratory syncytial virus strains were able to be differentiated by PCA using their PCA scores as shown in Figure 8A (Shanmukh et al., 2008). Recently, our group proposed a simple way to estimate compositions for binary and ternary chemical mixtures employing PCA and SERS (Hou et al., 2015a). As differences between the chemical compositions adsorbed on substrates and that in solution was neglected which is not suitable for most cases, we modified that method in order to provide a general route for the compositional analysis by considering the difference in chemicals' adsorption abilities in trace level solution (Zou et al., 2017). A standard mixture sample was introduced as the reference from which the adsorption kinetic factor of each component was obtained. Thus, predictions by PCA can be corrected by using these adsorption kinetic factors to eliminate the composition discrepancies between the substrates and the solution. As shown in Figure 8B, predictions for compositions in ternary solution were closer to their real value. Furthermore, the validity of this approach was demonstrated in binary, ternary, and quadruple chemical mixtures at trace level with the relative error of around 5%.

Partial least squares regression (PLSR) is another widely used chemometrics strategy for SERS analysis (Luo et al., 2016). In PLSR modeling process, intensities of all characteristic peaks were utilized and the redundant information would be eliminated by PCA. For example, Hou et al. reported quantitative analysis for single and binary food antiseptics by this strategy (Hou et al., 2016). Based on the regression relationship between potassium sorbate (PS) concentration and their corresponding SERS spectra, concentrations estimated by PLSR model were close to their actual value (Figure 8C). PLSR model was further employed for PS determinations in the binary solution of PS and sodium benzoate (SB). As shown in Figure 8D, predicted PS concentration values were around their actual values regardless of the different concentrations of SB in the measuring sample which contains the same concentration of PS.

## Applications

Advances in fabrications of Ag nanorods-based SERS substrates and the developments of quantitative analysis strategies have enabled SERS as a powerful technique to be utilized in various field. Herein, we highlight some SERS applications in the field of food safety, environment safety, biosensing, and vapor sensing.

### Food Safety

**Figure 9:**
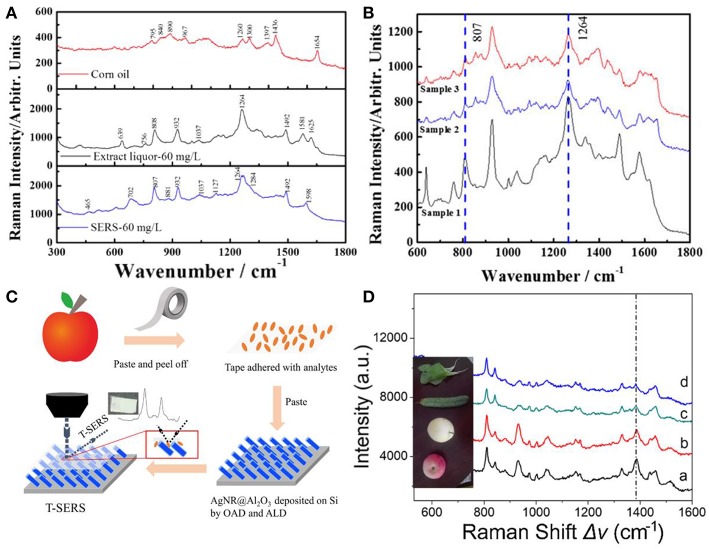
**(A)** SERS spectra of corn oil, the extract liquor from corn oil by methanol, and capsaicin. **(B)** SERS spectra of the extract liquor from gutter oil samples. Reproduced with permission from Tian et al. (2018). Copyright (2018) John Wiley and Sons. **(C)** Schematic representation for pesticide extracted from the curved samples. **(D)** SERS spectra of TMTD extracted from different fruits and vegetables. Reproduced with permission from Jiang et al. (2018). Copyright (2018) ACS publications.

Advantages of SERS, such as high sensitivity and easy operation have made it a powerful and promising technique for chemical detections in the field of food safety, such as beverages, pesticides, dairy production, and oil and so forth (Chen et al., 2015; Hou et al., 2016; Han et al., 2017; Roy et al., 2017; Jiang et al., 2018; Tian et al., 2018; Yao et al., 2018). For example, AgNRs substrate was employed for the rapid identification of gutter oil through capsaicin detection based on SERS (Tian et al., 2018). Figure 9A shows SERS spectra of the corn oil, extract liquor from corn oil which contains capsaicin, and capsaicin. The Raman bands from the extract liquor were similar to that of capsaicin, and distinct spectra differences between the corn oil and capsaicin were observed. Moreover, AgNRs substrate was employed to detect capsaicin in three gutter oils and Figure 9B shows their SERS spectra. Characteristic bands of capsaicin (807 and 1,264 cm^−1^) were clearly observed in gutter oils which demonstrates the potential to quantify capsaicin in gutter oil. Han et al. reported a detection limit of 0.3 mg/L for sodium saccharin by SERS (Han et al., 2017). They further employed SERS to detect the sodium saccharin in four soft drinks (sprite, cola, fanta, and schweppes) which were pre-extracted by ethyl acetate and obvious signals attributed to the sodium saccharin could be observed. Then, they estimated the ability of SERS to detect sodium saccharin in these four soft drinks with results far below the national standard, indicating the potential of SERS in practical applications. However, these detections rely on the analytes adsorbed from the solution to the surface of substrates, which are hard to be adopted for pesticide determinations on fruits and vegetables. Therefore, Jiang et al. developed an approach to allow SERS measurements on curved surfaces (Jiang et al., 2018). As shown in Figure 9C, analytes were transferred from the surface of apple to the surface of AgNRs-Al_2_O_3_ through the transparent and adhesive tapes. A high extraction efficiency of analytes (91.6%) was achieved in this way and evident SERS spectra of tetramethylthiuram (TMTD) extract from various vegetables and fruits can be observed in Figure 9D.

### Environment Safety

**Figure 10:**
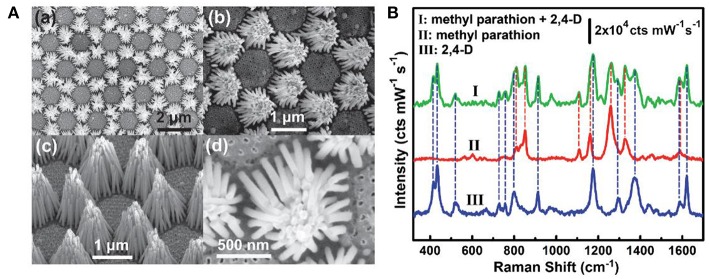
**(A)** SEM images of AgNRs bundle arrays. (a,b) Top views with different magnifications. (c) Side view. (d) Enlarged top view of a single AgNRs bundle. **(B)** SERS spectra of methyl parathion and 2,4-dichlo-rophenoxyacetic acid. Reproduced with permission from Zhu et al. (2016). Copyright (2016) John Wiley and Sons.

Environment pollutions pose great challenges to human health nowadays and many of these pollutants at trace levels are able to be rapidly detected on SERS platforms, such as the phosphates, metal ions, and phenolic compounds, and so forth (Ma et al., 2014; Ji et al., 2015; Zhu et al., 2016; Song et al., 2017; Wang et al., 2018; Li et al., 2019). For example, Zhu et al. fabricated a hierarchically ordered AgNRs array on the Au/Cu substrate through the binary template assisted electrodeposition method (Zhu et al., 2016). On the substrates, AgNRs bundles were hexagonally organized with each bundle consisting of 30–45 AgNRs. With the aid of the capillary force, small gaps were formed between adjacent AgNRs (Figure 10A) which created the high density of hotspots and exhibited about 10^8^ enhancement factor. Later, they estimated the ability of this chip to detect multiple pollutants in water and a mixture of methyl parathion (0.3 × 10^−6^ M) and 2,4-dichlo-rophenoxyacetic acid (2, 4-D) (2 × 10^−6^ M) was employed as the model molecules. As shown in Figure 10B, simultaneous detection of two analytes was achieved by observing different characteristic peaks of them which indicates SERS as a promising candidate in detection multiple organic pollutants in real world. Moreover, Song et al. developed a SERS sensor for Hg^2+^ in which AgNRs was modified with a single-strand oligonucleotide probe comprising of fifteen thymines (T) with a Cy5 dye labeled on the 3′-end and a thiol group at the 5′-end (Song et al., 2017). In the absence of Hg^2+^, the oligonucleotide probes were in flabby state, allowing the closest distance between the Cy5 dye and AgNRs and generating the strongest SERS signals. Upon Hg^2+^ addition, this single-strand oligonucleotide probe transformed into double stranded complex with the formation of T-Hg^2+^-T pairs, and this would keep the Cy5 dye away from AgNRs and decrease its SERS intensity.

### Biosensing

**Figure 11:**
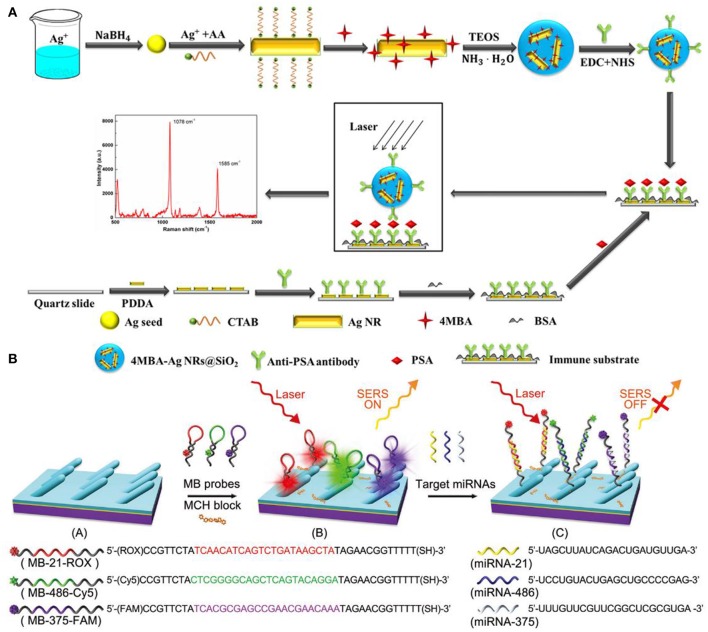
**(A)** Schematic illustration of the preparation for SERS-based immunoassay protocol. Reproduced with permission from Lai et al. (2015). Copyright (2015) RSC publications. **(B)** Schematic illustration of the preparation and measuring protocol for multiple miRNAs sensor. Reproduced with permission from Song et al. (2016). Copyright (2016) RSC publications.

As SERS provides abundant information in the molecular vibrational spectra with high sensitivity, it emerges as a powerful tool for biomolecules detections and diseases diagnosis (Lai et al., 2015; Chen et al., 2016; Song et al., 2016; Botta et al., 2018a; Zong et al., 2018). Immunoassays have been widely used for protein detections combining SERS. For example, Lai et al. utilized a sandwich immunoassay configuration which is composed of the immune probes/target protein/immune substrate to detect prostate-specific antigen (PSA) (Lai et al., 2015) (Figure 11A). In their work, silica-coated AgNRs aggregates with 4-mercaptobenzoic acid (4-MBA) molecules and AgNRs on slide substrates were both prepared and functionalized with anti-prostate specific antigen (anti-PSA) antibody to act as the immune probe and immune substrate, respectively. The sandwich detection system formed in the presence of PSA which remarkably boost the SERS intensity of 4-MBA molecules. Based on the correlations between PSA at different concentrations and characteristic SERS signals of 4-MBA, a limit of detection of 0.3 fg/ml was obtained. Moreover, the immunoassay protocol also exhibits good selectivity.

SERS also proves to be a powerful candidate in human diseases diagnosis. For example, Song et al designed a sensor for simultaneous detections of multiple cancer-related miRNAs in which AgNRs was modified with specific hairpin-shaped molecular beacons (MBs) (Song et al., 2016). As shown in Figure 11B, three kinds of MBs that contained different Raman reporter dyes and were complementary to miRNA-21, miRNA-486, miRNA-375 were prepared and modified on AgNRs via thiol group, leading the strongest specific SERS signals. After blocking the available regions for molecules on AgNRs by Mercaptohexanol (MCH), the sensor was employed for simultaneous detections of miRNA-21/486/375. Based on the signal reduction due to hybridization between MBs and the target analytes, the sensor exhibits excellent sensitivity and good specificity for the target analytes. Furthermore, because miRNA-21/486/375 were regarded as lung cancer-related biomarkers, the sensor was further utilized in patients' serum to achieve early detection of lung cancer. Ultra-sensitivity was obtained with the limits of detection of the three miRNAs are 393, 176, and 144 aM. Chen et al. reported the direct detection of malaria infected red blood cells by SERS (Chen et al., 2016). In their work, they observed distinct spectral differences between normal red blood cells (RBCs) and plasmodium falciparum infected RBCs (iRBCs) which were further analyzed by PCA to better illustrate the surface protein evolution for iRBC at different stages. In addition, the limit of detection for RBCs and iRBCs was measured in practical considerations and the ultimate sensitivity of a single cell can be reached based on the experiment results and theoretical calculation.

### Vapor Sensing

**Figure 12:**
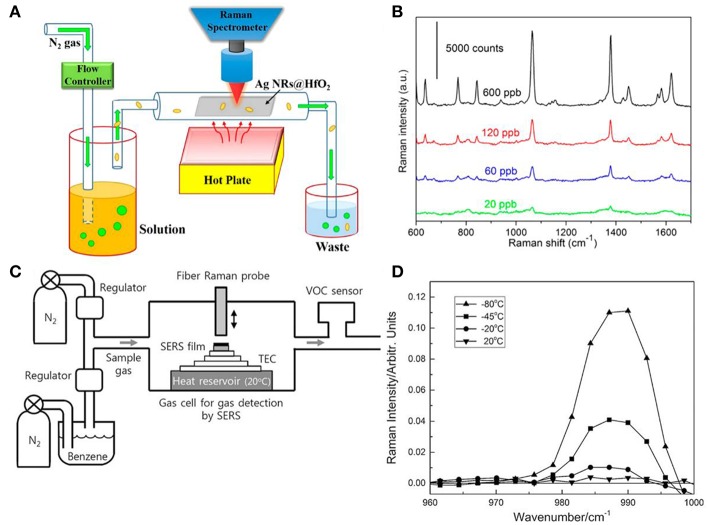
**(A)** Schematic illustration of real time SERS-based vapor sensing platform. **(B)** SERS spectra of 2-naphthalenethiol with various concentrations employing platform shown in **(A)** after 40 min gas flow. Reproduced with permission from Ma et al. (2016b). Copyright (2016) ACS publications. **(C)** Schematic illustration of the platform integrated with thermoelectric cooler (TEC) for benzene vapor detection. **(D)** SERS spectra of benzene gas with concentration of 34 ppm from modified AgNRs at different temperature. Reproduced with permission from Oh et al. (2018). Copyright (2018) John Wiley and Sons.

SERS can also expand its utilization from the aquatic environment to the vapor phase which should find applications in chemical sensing for volatile organic chemicals and explosives (Shah et al., 2013; Kreno et al., 2014; He et al., 2015; Ma et al., 2016b; Oh et al., 2018). Ma et al. designed a real-time gas sensing platform for 2-naphthalenethiol (2-NaT) and 2-mercaptopyridine (2-MPy) employing AgNRs-HfO_2_ substrates (Ma et al., 2016b). As shown in Figure 12A, 2-NaT and 2-MPy were dissolved in ethanol with various compositions which resulted in the different compositions of gas. AgNRs-HfO_2_ was placed into the sealed chamber and analytes in gas phase were carried by N_2_ gas to flow through the substrates. After exposure to 2-NaT gas for 40 min, SERS spectra of 20 ppb 2-NaT could be observed on substrates as shown in Figure 12B. Moreover, the mixture vapor gas of 2-NaT (600 ppb) and 2-MPy (600 ppb) was successfully recognized utilizing this platform which indicates its effectiveness for detections of vapor molecules. Similarly, Shah et al. reported a strategy for vapor detection of 4-aminobenzenethiol (4-ABT) (Shah et al., 2013). Due to the highly volatile of 4-ABT and its thiol group, 4-ABT vapor for different exposure times were successful detected on AgNRs. However, some molecules are weakly adsorbed on plasmonic materials, making it a difficult task to detect them. Thus, various molecules modifications on plasmonic materials were explored by researchers, such as metal–organic frameworks and thiol compounds (Lee et al., 2019). For example, Oh et al fabricated AgNRs and functionalized it with propanethiol (Oh et al., 2018). Due to the physical adsorption of benzene molecules to propanethiol, SERS spectra of benzene in vapor phase were successfully observed on substrates and the detection ability of this sensor was found ~1 ppm at room temperature with 120 s acquisition time. To further decrease the limit of detection, they designed a SERS-based benzene gas detection apparatus in which thermoelectric cooler was integrated for the purpose of deep cooling of substrates (Figure 12C). SERS spectra of benzene gas with concentration of 34 ppm were obtained from modified AgNRs at different temperatures. As shown in Figure 12D, lower temperature for substrates enhanced Raman intensity of benzene obviously. They further found that sensitivity of the gas sensor for benzene greatly improved from ppm to ppb level when the substrate was cooled down from room temperature to −80°C, and they attributed this great sensitivity improvement to the enhancement of the amount of gas adsorption resulted from the cooling effect of substrates.

## Conclusions and Perspective

In this review, we introduce advancements in AgNRs preparation through OAD technique that help guide rational manipulation of SERS substrates to acquire superior sensitivity, high uniformity, and excellent reproducibility. Considering the intrinsic fatal drawbacks of AgNRs, our review covers approaches to integrate oxides with AgNRs with the aim to endow SERS substrates with better stability and recyclability and to allow SERS technique be suitable for a broader application scope. Moreover, accompanied by these significant progress in substrates fabrications, three different quantitative analysis strategies were developed and we discuss them in detail to gain a reasonable and accurate data analysis. Furthermore, we highlight SERS applications in the fields of food safety, environment safety, biosensing, and vapor sensing, demonstrating the potential of SERS as a versatile and promising technique.

Although remarkable progress have been made on substrates to pursue better SERS performance and various quantitative analysis strategies were established to obtain reliable results, many challenges still remain to be addressed. First, despite that thriving researches in substrates manipulation enable the realization of improved SERS sensitivity down to even single-molecule level, single molecule detection sensitivity in complex matrix especially the biological system remains challenging. Trapping the molecules of interest to the hotspots by surface modification of substrates emerges as a promising strategy to solve this issue as it can simultaneously enhance SERS detection sensitivity and avoid incorrect SERS information brought by the interference molecules. However, effective surface modification approaches are still limited. Therefore, further advancement in the surface modification to precisely control analytes close to the hotspots are expected.

The bi-functional hybrid that incorporating catalysts (Au, Pd) with AgNRs-based substrates combines the merits of the abundant molecules information brought by SERS and catalytic activities provided by the catalyst, enabling it as a promising platform to monitor catalytic reactions *in situ* and to elucidate the catalytic mechanism. However, current studies related to this field are mainly focused on the hybrids manipulations to obtain optimized SERS sensitivity and catalytic activity, and SERS monitoring for catalytic reactions are limited to only a few model systems, such as reduction of 4-nitrothiophenol to 4-aminothiophenol and oxidation of 4-aminothiophenol to trans-4,4′-dimercaptoazobenzene. Therefore, more catalytic reactions monitored by SERS need to be explored to help uncover the reaction mechanism and to fully exploit the potential of bifunctional hybrid systems. Moreover, while employing SERS as a tool to monitor dynamic processes including catalytic reactions and biological processes, high time resolution is of great importance to provide detailed information about the process, especially for reactions occurred at a time scale of ms or dynamic SERS measurement of a living cell. Thus, more efforts are needed to improve the time resolution of SERS technology.

## Author Contributions

SZ and ZZ wrote the manuscript. LM, JL, YL, and DZ provided critical feedback and helped revision of the manuscript.

### Conflict of Interest Statement

The authors declare that the research was conducted in the absence of any commercial or financial relationships that could be construed as a potential conflict of interest.
